# Synthesis of large scale 3D microscopic images of 3D cell cultures for training and benchmarking

**DOI:** 10.1371/journal.pone.0283828

**Published:** 2023-03-31

**Authors:** Roman Bruch, Florian Keller, Moritz Böhland, Mario Vitacolonna, Lukas Klinger, Rüdiger Rudolf, Markus Reischl

**Affiliations:** 1 Institute for Automation and Applied Informatics, Karlsruhe Institute of Technology, Eggenstein-Leopoldshafen, Germany; 2 Institute of Molecular and Cell Biology, Mannheim University of Applied Sciences, Mannheim, Germany; Yeungnam University, KOREA, REPUBLIC OF

## Abstract

The analysis of 3D microscopic cell culture images plays a vital role in the development of new therapeutics. While 3D cell cultures offer a greater similarity to the human organism than adherent cell cultures, they introduce new challenges for automatic evaluation, like increased heterogeneity. Deep learning algorithms are able to outperform conventional analysis methods in such conditions but require a large amount of training data. Due to data size and complexity, the manual annotation of 3D images to generate large datasets is a nearly impossible task. We therefore propose a pipeline that combines conventional simulation methods with deep-learning-based optimization to generate large 3D synthetic images of 3D cell cultures where the labels are known by design. The hybrid procedure helps to keep the generated image structures consistent with the underlying labels. A new approach and an additional measure are introduced to model and evaluate the reduced brightness and quality in deeper image regions. Our analyses show that the deep learning optimization step consistently improves the quality of the generated images. We could also demonstrate that a deep learning segmentation model trained with our synthetic data outperforms a classical segmentation method on real image data. The presented synthesis method allows selecting a segmentation model most suitable for the user’s data, providing an ideal basis for further data analysis.

## Introduction

The automated analysis of image and video streams from microscopes has become well established for the assessment of, e.g., cell states. Conventional image processing methods allow for the generation of single features and time series (live cell imaging), which are subsequently used for state estimation of cells.

**Fig 1:**
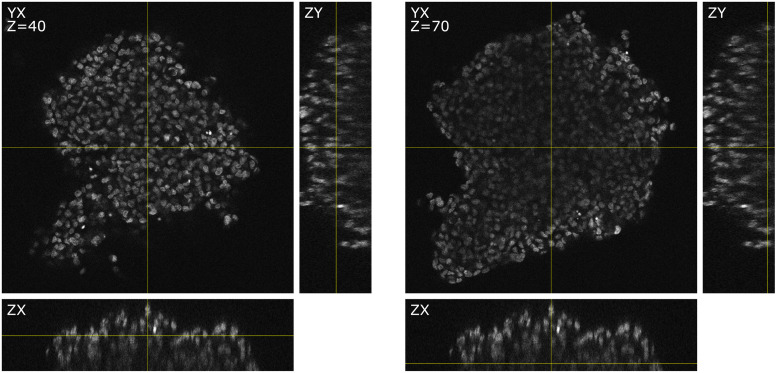
Orthogonal image slices of a 3D cell culture recording. The images are grouped in two triple-sets, each displaying *xy*, *zy* and *zx* views of the same data set. The left side shows a slice at the *z*-depth 40, and the right side shows a slice at the *z*-depth 70. The images show a varying quality dependent on the region and a reduced sharpness in the *z*-axis as a result of the PSF being more spread out in this direction.

Advancing biotechnology enables the generation and imaging of complete 3D cell cultures such as spheroids or organoids. Such structures are often preferred in biological studies because they provide a cell environment more similar to the human organism than conventional mono-layer cell cultures [1, 2]. However, this advantage comes with its drawbacks. The three-dimensional structure leads to a constant change of organic material along the *z*-axis, differing in their refractive index. This increases light scattering in deeper regions, reducing the sharpness and brightness of the image. Fig 1 shows two 2D slices of the same 3D image and their corresponding orthogonal views. The left and right images show a depth of *z* = 40*px* (60*μm*) and *z* = 70*px* (105*μm*), respectively. While the image quality of the upper part (left image) is relatively uniform, the lower part (right image) shows areas of varying brightness and image quality. Outer regions show less deterioration since fewer occluding structures are located above them. In addition, a typical limitation of confocal microscopy can be seen in the orthogonal views: the achievable resolution in the *z*-axis is much lower compared to the *y*- and *x*-axis, as the point spread function (PSF) is more spread out in this axis. Furthermore, the increased blurring of the structures in this direction also lets objects appear bigger than their actual size.

Machine-learning approaches, specifically deep-learning methods, have proven powerful in the medical and biological domains under different conditions [3–7]. However, these methods require large annotated data sets for training and subsequent evaluation to achieve this performance [6, 8, 9]. While the annotation of 2D data is time-consuming and burdensome, the annotation of 3D data is an almost impossible task. The additional dimension massively increases the amount of data to be annotated. In addition, the limitation to visualizing 3D structures in 2D images makes it difficult to recognize volume structures and leads to inconsistent annotations between layers. Additionally, unsharp object borders in *z*-direction make start and end points of objects hard to define. The varying quality and anisotropic resolution thus make accurate and consistent labeling of the structural outline impossible.

An alternative approach to the manual annotation of training data is the synthesis of real recordings, where the labels are known by design. The methods can be divided into *simulation-based* and *data-driven* approaches. In simulation-based approaches, parametric models are used to reproduce real characteristics, while in data-driven approaches, algorithms learn to reproduce real images based on the data itself. Hybrid models combining both approaches in different steps are also possible.

A common simulation-based tool for generating synthetic cell nuclei images is CytoPacq [10]. Due to the complex simulation, the generation of large images containing a high number of nuclei is time-consuming in this tool. Additionally, the online version only allows the placement of a limited number of nuclei. Since the program was not designed to generate large 3D cell structures, relevant characteristics like the deterioration of the image quality in deeper layers are not simulated. Stegmaier et al. [11] introduced a simulation-based method for the synthesis of 3D+t cell data. The main component of the method is a simulation of the nucleus positions and the corresponding cell stages. First, cell nuclei are extracted from synthetic time-lapse images and inserted in an empty image. In the subsequent acquisition simulation, a depth-dependent linear brightness decay, a point spread function, and noise are applied to the image.

Data-driven approaches mainly rely on generative adversarial networks (GANs) [12], where two networks are trained in a competitive manner. A generator network attempts to generate data samples to fool a discriminator network, which in turn attempts to distinguish the generated samples from real samples. The introduction of the Cycle-GAN [13] enables the use of unpaired images to learn the transformation between two image domains, i.e., no matching image pairs between the two domains are needed. For the transformation between label images and real images, one no longer needs exact segmentations corresponding to the real images, but random labels can be used.

Fu et al. [14] applied the Cycle-GAN to create synthetic training data for a 3D segmentation network. Synthetic label images are generated and afterwards transformed for the synthesis of microscopic images. Due to mismatches in the spatial correspondence, an enhanced version, the spatially constrained Cycle-GAN, is introduced. A disadvantage of their method lies in the 2D-based transformation. The 3D label image is transformed layer by layer and afterwards reassembled, which can lead to hard transitions between the layers. Dunn et al. [15] use synthetic data generated with the spatial constrained Cycle-GAN to train a deep learning segmentation network. It is shown that the network can outperform classical segmentation methods on heterogeneous data. Han et al. [16] later tested the Recycle-GAN [17], developed for the transformation of video data, to improve the transitions between layers caused by the 2D transformations by treating the third dimension as the time axis.

Eschweiler et al. [18] use an adapted Cycle-GAN to transform label images of cell membranes into real images. The transformation is also limited to 2D data. During training, a structure-aware loss is used to ensure consistency between the label and image data. They also introduced multiple parametric models to generate synthetic cell membrane labels. Later, Eschweiler et al. [19] introduced a 3D synthesis method based on a conditional GAN. As positional information would be lost due to the required patch-based transformation, they propose a conditional input to encode the positional information of each voxel. They further claim that the positional encoding function can also control the quality of the generated synthetic images. While this seems to be true for noise and sharpness, the brightness distribution seems to be mostly unaffected by the encoding. In order to reduce tiling artifacts, an advanced overlapping strategy is utilized. However, averaging overlapping areas leads to a reduction in noise.

The current state of the art simulation models suffer from one of two problems. Either the simulation method is so complex that cumbersome and often non-intuitive parameterization together with long calculation times are required, or the simulation method is simplified and therefore does not produce as realistic data. Furthermore, there is no simulation method that realistically models the reduced brightness and quality in deeper image regions.

Current data-driven approaches are mostly limited to 2D transformations and are not able to generate large 3D images with realistic global properties. Only the method introduced by Eschweiler et al. [19] enables the generation of such 3D images. However, this method relies solely on the identity loss to achieve structural correlation between labels and synthetic data. To avoid structural discrepancies, they put great effort into the generation of label data used to train the GAN by utilizing manual annotations or automatic segmentation.

Both, simulation-based and data-driven approaches have specific weaknesses. Therefore, we develop a new method for the synthesis of large 3D spheroid data which combines simulation-based and data-driven learning methods (3D Cycle-GAN) into a new hybrid approach. This allows the use of a simplified simulation while still producing high-quality results. In contrast to existing data-driven methods, simulated data is used for the training of the GAN. This reduces the domain gap and thus minimizes the risk of undesired discrepancies introduced by the transformation. Furthermore, we introduce a new brightness reduction model that depends on the image content to realistically model the brightness decay in deeper image regions. A new measure is presented to evaluate the results of the introduced reduction model. Furthermore, two measures are used to compare the generated synthetic data to the real data. Finally, the real data segmentation performance of a deep learning segmentation model trained with generated synthetic data is tested.

With the synthetic images and labels generated by our method, users can create large datasets for training 3D deep learning segmentation models. The synthetic data furthermore allows the selection of a segmentation model most suitable for the data to be analyzed. Based on the segmentation of real data, detailed analysis, like the correlation with additionally stained markers, can be performed.

## Materials and methods

### Concept

Formerly, the process of data segmenting consisted of selecting a segmentation algorithm and tuning its parameters. However, as deep learning methods became popular, they mostly replaced traditional segmentation algorithms. The process then consisted of selecting an appropriate deep-learning network and manually creating enough training data. Nowadays, the trend is to synthesize training data, again changing the process of data segmentation. First, a deep learning network is selected, and then labeled synthetic training data is created to train the selected model. As the quality of the generated training data greatly impacts the subsequent segmentation performance, sophisticated synthesis methods are required.

**Fig 2:**
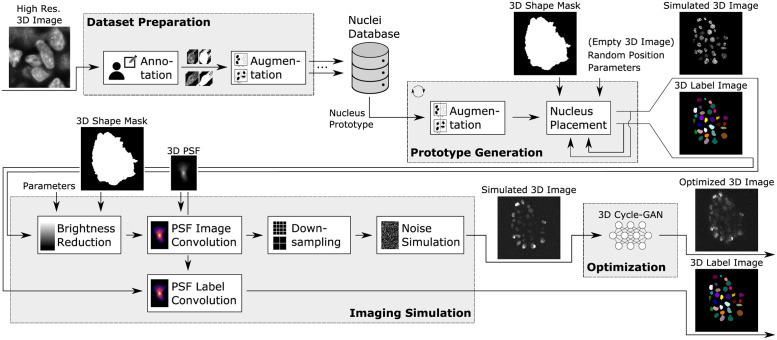
Concept of the data synthesis pipeline. The pipeline consists of four steps: dataset preparation, prototype generation, imaging simulation, and optimization. In favor of a clear visualization, 3D images are shown as 2D slices.

As mentioned, there are two main ways to generate labeled synthetic data: *simulation-based* and *data-driven*. Simulation approaches either suffer from a simplified model generating low-quality data or a long runtime if a complex simulation model is used. On the other hand, data-driven approaches mainly use GANs to transform binary label data into real-looking images. However, such networks often suffer from inconsistencies introduced by the transformation if the label data does not correspond to the real data [20–22]. For example, additional cells can be placed during the transformation in case of differing cell densities or changed nuclei shapes if ellipsoids are used as label data. Therefore, significant effort is needed to generate label data that matches the properties of the real data. We want to overcome this issue by drastically reducing the domain gap using simulated data instead of binary label data. For the synthesis of 3D microscopic images, we therefore introduce a hybrid approach based on parametric simulation and data-driven optimization. The procedure can be divided into four parts (see Fig 2): dataset preparation, prototype generation, imaging simulation, and optimization. A detailed description can be found in S1 Appendix.

The pipeline relies on a database containing 3D nucleus images and labels. The data for this *nuclei database* is generated during the *dataset preparation*. Typically a small number of nuclei is sufficient, which can be manually extracted from real images. Ideally, deconvoluted images with high resolution are used for this task, as the influence of the microscope optics is minimal here. In addition, the individual nuclei are easier to annotate in such images. Once the user has annotated the nuclei, augmentation (rotation, scaling, blur, contrast, gamma, and elastic transformation) is used to further increase the number of nuclei in the database.

The *nuclei database* is then used during *prototype generation* to generate a *simulated 3D image* showing a 3D cell culture. First, a *nucleus prototype* is randomly drawn from the *nuclei database*. An additional basic *augmentation* procedure further increases the variability of the nucleus samples. In contrast to the augmentation in the *dataset preparation*, this augmentation is applied on each repetition, making a low runtime crucial. Therefore, only random flipping and axis swapping are used. During the following *nucleus placement*, a random position is sampled and checked for its validity. This ensures that overlaps with other nuclei are avoided and that the position lies within an optionally provided shape mask, enabling the generation of a cell culture with a realistic 3D shape. After the validation of the position, the first nucleus is placed in an empty 3D image.

Following the placement of the first nucleus or in case of an invalid position, the process is repeated by drawing a new random nucleus prototype from the *nuclei database*. This process is then repeated until a given number of consecutive attempts to place new nuclei is reached. In this case, the process is stopped, and the *prototype generation* is completed. The number of consecutive attempts therefore influences the uniformity of the generated sample. With a lower number (e.g., 50), holes will appear inside the cell culture. A higher number (e.g., 2000) will lead to an evenly filled cell culture. The cell density of the generated cell culture can also be adjusted with the parameters of the *nucleus placement*. The output of the *prototype generation* is a *simulated 3D image* of a cell culture sample and its corresponding *3D label image*.

In *imaging simulation*, the recording process of the microscope is simulated and applied to the image. One important aspect of confocal imaging is the reduced brightness and quality in deeper regions of the sample. This effect is modeled by the *brightness reduction*: for each voxel (*z*, *y*, *x*) in the synthetic image, a brightness reduction factor *b*(*z*, *y*, *x*) is calculated. The factor depends on *i*(*z*, *y*, *x*), the number of foreground voxels above (*z*-direction). The foreground is defined by the shape mask also used in the *prototype generation*. As different optical clearing methods can vary in effectiveness [23], the reduction can be tuned by the parameter *p*. In summary, the brightness reduction factor *b* is calculated as follows:

$$\begin{array}{c} b\left(z,y,x\right)=max(min\left({\left(\frac{i(z,y,x)}{p}+1\right)}^{-6},1\right),0). \end{array}$$ (1)

The intensity of each voxel in the synthetic image is then multiplied by the brightness reduction factor *b*.

Following the *brightness reduction*, the blurring effect of the diffraction-limited optics is simulated by the *PSF image convolution*. In this step, the image is convolved with multiple point spread functions to generate a reduced quality in deeper image regions. The convolution increases the perceived size of the nuclei. Labels created by manual annotation would therefore be larger in size compared to the generated ones. To optionally mimic this effect, the size of the labels can be increased by a *PSF label convolution* utilizing the same point spread functions.

After the convolution, *downsampling* can be applied to the image to match a specific output resolution. The downsampling mimics the discretization effect by the microscope’s image sensor. The downsampling also allows the PSF convolution to be performed in higher resolution, increasing the quality. Afterwards, the *noise simulation* calculates and applies Poisson noise to the image.

Since not all microscope effects can be modeled in full detail in the imaging simulation, the generated *simulated 3D images* differ slightly from the real ones. For example, the noise within the cell culture is less pronounced than in real images. We therefore utilize a *3D Cycle-GAN* as an optimization method to reduce such differences. For this, the Cycle-GAN receives the *simulated 3D image* as an input and transforms it to the real domain, resulting in the *optimized 3D image*. In contrast to other methods directly transforming binary label masks, the use of simulated data reduces the domain gap. This minimizes the risk of the transformation introducing unwanted differences between domains, such as the placement of additional nuclei not present in the label maps [20–22].

Since Cycle-GANs are heavy on GPU memory, the images need to be split into smaller patches before the transformation. This is not ideal since regional information is lost in this process, and the independent transformation can lead to different image properties in neighboring patches. Other methods therefore use an auxiliary conditional layer containing regional information. However, as we use simulated data instead of binary labels, global information like the brightness distribution is directly contained in the patches. This leads to a more consistent transformation, minimizing the differences between neighboring patches after their individual processing.

After the image patches are transformed by the 3D Cycle-GAN the *optimized 3D image* is reassembled from the individual patches. Border regions of transformed patches can contain artifacts due to missing information in convolution operations. As this can lead to hard transitions between the patches, the border regions of the transformed patches are omitted by using overlapping patches.

In the following, the term naive is used to describe data generated with the presented simulation pipeline, i.e., without the optimization step.

### Synthetic data generation

This section describes the parameters used to generate the naive and optimized image data. In total, four images were generated. The shape masks for the images were generated with the automatic mask generator of the pipeline. To fill the *nuclei database*, a total of ten nuclei were manually segmented as described in Data set and augmented to create a total number of 320 nuclei. he number of consecutive placement attempts for the *prototype generation* was set to 1000, and no overlap between the nuclei was allowed. Furthermore, no optional enlargement of nuclei was used to increase the nuclei distance in the culture.

The brightness reduction parameter *p* was set to 150, and a single measured PSF was used for the *PSF image convolution*. The simulation was performed with a voxel size of 0.9 × 0.122 × 0.122 *μm*^3^(*z*, *y*, *x*), which was increased to 1.5 × 0.489 × 0.489 *μm*^3^(*z*, *y*, *x*) in the *downsampling* step. After the downsampling, Poisson noise was calculated and applied to the image.

The training data for the optimization network were generated from four real and four naive images. The network was trained on a *NVIDIA RTX A6000* for 7000 epochs requiring a time of 145*h*. A detailed description of the network structure and training/inference procedure can be found in S1 Appendix.

### Quality evaluation measures

Evaluation criteria are necessary to check the quality of the generated synthetic images quantitatively and to compare them with real-world images. As the position of nuclei differs between real and synthetic images, standard evaluation measures like the mean squared error or the structure similarity index (SSIM) cannot be applied. In this section, we introduce three measures for comparing our data. The *Wasserstein distance* calculates the difference between intensity distributions of generated and real images. The *q95 edge quality* explicitly compares the quality and brightness reduction in increasing image depths. Finally, the DET measure [24] is used to test the real-world performance of segmentation models trained on synthetic data.

#### Wasserstein distance

An intuitive way to compare real and synthetic images is to analyze their intensity histograms. Histograms are frequently used to visualize the intensity distributions of images. They can thus give information about the noise distribution or how well the signal is separated from the noise.

As a visual comparison of histograms is not suitable as a quantitative measure, the Wasserstein distance [25] is used to calculate the difference between two distributions. To obtain a more informative output, we propose a novel division of the analyzed images into three regions: the background outside, the background inside, and the foreground inside the cell culture. Importantly, this split avoids the dependency of the measure on the ratio of foreground to background signal, e.g., the cell density.

To define the regions, a corresponding label mask is needed. For the naive and optimized images, the generated label mask is given. For the real images, the segmentation network trained with synthetic data is used to calculate the label masks. The detailed procedure for calculating the three image regions is described in S2 Appendix.

The Wasserstein distance *W* is calculated between the real and naive as well as between the real and optimized images. The distance is calculated for each image region and each *z*-slice separately. Finally, the difference between the real and a completely black image is calculated to normalize the Wasserstein distance *W* to an output range of [0, 1]. This difference *W*(*p*_real_, *p*_black_) is seen as the worst possible result. The normalized distance metric *W*_norm_ is then defined as:

$$\begin{array}{c} W_{\text{norm}}\left(p_{\text{real}},p_{\text{syn}}\right)=1-\frac{W(p_{\text{real}},p_{\text{syn}})}{W(p_{\text{real}},p_{\text{black}})}. \end{array}$$ (2)

The distribution of either the naive or the optimized image is given by *p*_syn_. With the normalization, a value of 1 indicates the best possible result. It is obtained if the compared distributions are the same.

#### Edge quality

The reduced quality and brightness in deeper regions of a sample is an essential aspect of 3D microscopic imaging. Therefore, a new measure is introduced to measure and compare how well this feature is represented in the generated synthetic data.

The primary source of the deterioration results from light scattering enlarging the PSF, leading to washed-out structures and a lower sharpness. This can be quantified by the edge strength of the present image structures. Therefore, the introduced measure for quality deterioration is based on the Sobel edge filter. A 95% quantile is used to summarize the results of the edge image into a single value:

To extract the edges of the image, each *z*-slice of the input image is first smoothened by a Gaussian filter (*σ* = 3) and subsequently filtered with the Sobel operator. The high *σ* value ensures that the resulting edge image is not influenced by noise but focuses on the difference between foreground and background intensity.

As 3D spheroid cultures are approximately round in shape, objects in the same *z*-slice are less occluded if they are located in outer regions. A comparison of complete slices is therefore not sensible. Thus, the edge image is divided into multiple *z*_*n*_ × 100 × 100 *px*^3^ sections, where *z*_*n*_ indicates the number of *z*-slices of the image. For each *z*-slice *i* in a section *j*, the 95% quantile *e*_0.95,*j*, *i*_ of the edge image is calculated.

Due to the roundish shape of the cell cultures, the foreground in each section will start at a different image depth, making a *z*-axis alignment between the sections necessary. The normalized *z*-index *d*_*j*_ is calculated based on the *z*-index with the highest value of *e*_0.95,*j*, *i*_:

$$\begin{array}{c} d_{j}=i-\underset{i}{\text{arg max}}e_{0.95,j,i}. \end{array}$$ (3)

Sections with a maximum value smaller than a threshold *t*_*b*_ are discarded as they only show background signals. Equally discarded are sections whose slice index of the highest value is greater than *t*_*r*_. The number of remaining sections is given by *s*_*n*_. Finally, the q95 edge quality measure is calculated for each normalized *z*-index *d*:

$$\begin{array}{c} e_{0.95,d}=\frac{1}{s_{n}}\sum_{j=1}^{s_{n}}e_{0.95,j,d_{j}}, \end{array}$$ (4)

where *s*_*n*_ represents the number of remaining sections.

#### Segmentation

A central goal of synthetic data generation is the training of deep learning segmentation models which can then be used to segment real data. The closer the synthetic data is to the real data, the better segmentation results can be expected. We therefore train 3D segmentation networks with the synthesized data and test their performance on real images. As a basis, we use the model proposed by Scherr et al. [7]. The network structure is adapted to allow for direct 3D segmentation. As training data, image patches with the size of 32 × 128 × 128 *px*^3^ (*z*, *y*, *x*) are used. Due to the increased memory usage of 3D processing, the batch size is reduced to three. The Ranger optimizer is used in combination with an initial learning rate of 1*10^−3^. The remaining training settings are consistent with those used in [7].

Three models are trained. The first model *S*_naive_ is trained with only naive data, i.e., generated by the proposed method without the optimization step. The second model *S*_opti_ is trained with only optimized data, i.e., the same data as for the first model, but optimized with the 3D Cycle-GAN. The third model *S*_naive+opti_ is trained with both naive and optimized data. The models *S*_naive_ and *S*_opti_ are trained with two synthetic spheroid images, and one additional image is used for training validation. For the third model, *S*_naive+opti_, four images are used for training and two for training validation.

Furthermore, the conventional segmentation method TWANG [26], implemented in XPIWIT [27], is used to enable a comparison of the deep learning models. Before the segmentation, the images are filtered by a Gaussian smoothing operator with *σ* = 1. The pixel spacing for *x* and *y* are set to 1, and spacing in *z* is set to 2.64 to match the image data. Based on manual nucleus diameter measurements, *σ*_*min*_ and *σ*_*max*_ values for seed detection are set to 4 and 9, respectively, with a step size of 0.5. The key point threshold for the seed detection is tuned manually to achieve the best performance on the DET score (described later) and is set to 0.15. Otherwise, the parameters are left unchanged from the ones in the provided example.

To measure the performance of segmentation models, ground truth data of real images is needed. As the labeling of real 3D data is nearly impossible, the ground truth creation is reduced to a center point annotation task. A real image region of 32 × 256 × 256 *px*^3^ (*z*, *y*, *y*) is annotated using the 3D Slicer [28] software. The annotations are performed by a person with labeling experience but limited biological expertise. Afterwards, the labels are verified by a biological expert to ensure their correctness.

To evaluate the performance of the models based on the center point annotations, the detection measure introduced in [24] and provided by the cell tracking challenge [5] is used. The measure provides a combined detection accuracy value. Additionally, the number of false positives, false negatives, and missed splits are determined, allowing the calculation of the final detection accuracy. The number of false splits is not explicitly given by the measure, but is rather included in the false positive count.

### Data set

To generate synthetic microscopic images of large 3D cell cultures, a hybrid approach, combining classical simulation and data-driven optimization is used. The data presented in this section serves as the basis for the development of the synthesis pipeline. It is furthermore used as training data for the data-driven optimization and to evaluate the similarity of the synthesized data to the real data. We want to emphasize that no special cell culture protocol is required for image synthesis with the proposed method.

As real-world data, spheroid cultures of MDA-MB-231 human breast cancer cells, grown in 96 well plates, are used. The spheroids are stained with DAPI. Imaging was performed with a confocal microscope (Leica Microsystems, Mannheim, Germany; TCS SP8) with an HC PL APO 20 x/0.75 objective. Images were taken at a resolution of 1024 × 1024 *px*^2^ with a step size of 1*μm*. This results in a voxel size of 0.45 × 0.061 × 0.061 *μm*^3^ (*z*, *y*, *x*). The images range in their *z*-depth from 77 to 120 *px*. Further information about the protocol is provided at [29].

For the extraction of nuclei prototypes, one spheroid was separately recorded with a higher magnification to obtain a voxel size of 0.45 × 0.061 × 0.061 *μm*^3^ (*z*, *y*, *x*). This image was deconvolved using the DeconvolutionLab2 ImageJ plugin [30]. Afterwards, ten individual nuclei are annotated using the 3D Slicer [28] software by a person with labeling experience but limited biological expertise.

A data set containing four real and four naive 3D images is used to train the aforementioned optimization. The naive images are created with the same resolution and cell culture shape as their real counterparts. Due to the generation process of the naive images, the positions of the nuclei differ from those of the real images. The same four real and synthetic images are used for the quality evaluation as described in the methods.

### Statistical analysis

The later shown results of the normalized Wasserstein distance and the q95 edge quality measure are statistically analyzed, as described in the following. The results of naive and optimized images for each region and z-slice are compared. As the optimized images are based on naive images, and both are compared to the same real images, the samples are considered to be paired.

To test whether the requirements of the t-test are fulfilled, the distribution of the samples is analyzed. Based on the central limit theorem, the result samples of the q95 edge degradation measure should follow a normal distribution. However, in some cases, the distribution visually differs from the normal distribution. This finding was verified using the Shapiro–Wilk test, which shows that the null-hypothesis (the data is normally distributed) can be significantly (p<0.05) rejected in five of six cases. In the case of the normalized Wasserstein metric, the null-hypothesis of this test can be rejected in 14 of 18 cases. Therefore, the nonparametric Wilcoxon signed-rank test is used with the one-sided alternative hypothesis to compare the results of the two measures statistically. All p-values are calculated using the exact method. Compared to the t-test, the Wilcoxon signed-rank test is more conservative. The statistical analysis was performed using the python package *scipy* with the functions *scipy.stats.shapiro* (Shapiro-Wilk test) and *scipy.stats.wilcoxon* (Wilcoxon signed-rank test).

## Results

### Intensity distributions

**Fig 3:**
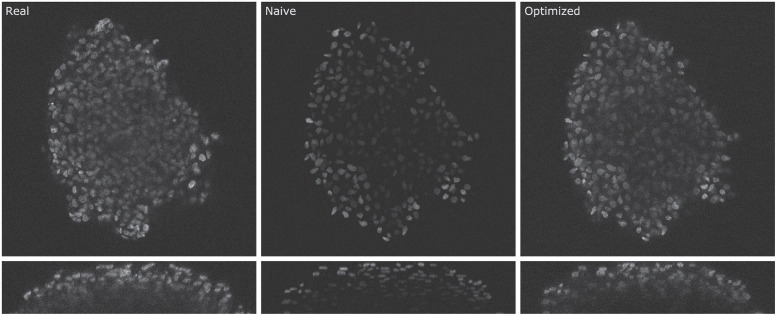
2D slice of a real, naive and optimized image (top) and corresponding enlarged orthogonal views (bottom). While the shape of the cell culture roughly matches between real and synthetic data, the positions of the nuclei are different due to the generation process of the synthetic data.

For a visual comparison between real, naive, and optimized images, corresponding 2D slices are shown in Fig 3. The upper row shows *x* − *y* and the lower row *y* − *z* slices. Inside the spheroid, the naive image looks sharper and less noisy than the real image. Especially in the darker center region, the simulation misses the increased signal spreading of the real data. In the side view, the simulated nuclei look smaller and more flat compared to the real data. The optimized image shows considerably more noise inside the spheroid region and a decreased sharpness compared to the naive image, which renders it more realistic. The nuclei in the side view appear larger and less flat compared to the naive data. The shapes of the nuclei in the optimized image mostly match the ones in the naive image, although they appear more blurred. Only in a few cases does a nucleus’s shape slightly differs between naive and optimized image. There seem to be no object mismatches, e.g., additionally placed or removed objects. The shape of the synthetic cell culture is nearly identical to the real counterpart. Furthermore, the brightness reduction of the real image is well represented in both the naive and the optimized image. As in the real image, the nuclei brightness does not depend on the distance to the border but rather on the amount of structure above.

**Fig 4:**
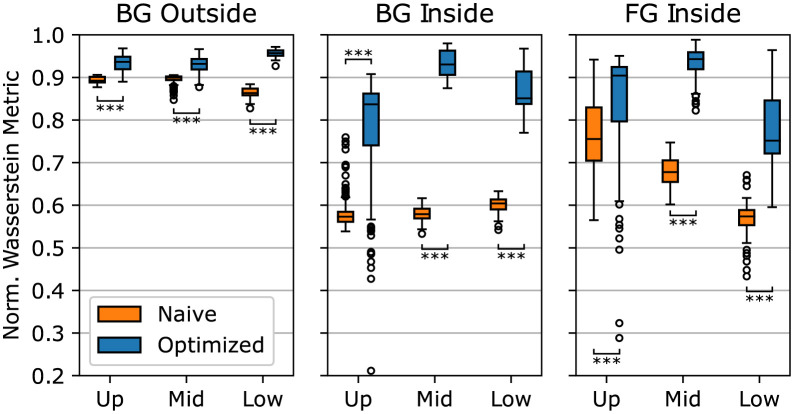
Normalized Wasserstein metric between intensity distributions of real and naive (orange bars, Naive) and real and optimized data (blue bars, Optimized). The measure is calculated for three image regions: background outside and inside the spheroid, and foreground. The images are divided into an upper, middle and lower part. A value of one indicates a perfect result, while a value of zero indicates a bad result.

The results of the Wasserstein distance analysis are shown in Fig 4. The normalized distance of naive and optimized images are given for three image regions: background outside and inside the cell culture and foreground. Additionally, the *z*-slices are grouped into the regions upper, middle, and lower with intervals of [0, 41), [41, 82), and [82, ∞) *px*, respectively.

The difference between the real and naive data is the smallest for the background region outside the spheroid. The median scores for the upper, middle, and lower image parts are 0.89, 0.9, and 0.86, respectively. The optimization of the naive data significantly improves the score with median values between 0.94 and 0.96 (exact Wilcoxon test: *w* = 46, *p* <.001, *n* = 156 ∣ *w* = 195, *p* <.001, *n* = 157 ∣ *w* = 0, *p* <.001, *n* = 48).

The overall largest difference between real and naive data exists in the background region within the spheroid. In this region, the median values lie between 0.57 and 0.6. However, the optimization significantly improves the score for this region with lowest and highest median values of 0.84 and 0.93 (exact Wilcoxon test: *w* = 545, *p* <.001, *n* = 148 ∣ *w* = 0, *p* <.001, *n* = 156 ∣ *w* = 0, *p* <.001, *n* = 45). The larger scatter of the results, especially in the upper part of the image, is noticeable.

While the naive data in the two background regions show relatively consistent results between the image’s upper, middle, and lower portions, the results for the foreground region worsens for deeper image sections. The median values in this region are 0.76, 0.68, and 0.57 for the upper, middle, and lower image parts, respectively. The scatter of the individual results is larger compared to the two background regions. The optimization also significantly reduces the difference to the real data in the foreground region (exact Wilcoxon test: *w* = 3593, *p* <.001, *n* = 164 ∣ *w* = 0, *p* <.001, *n* = 159 ∣ *w* = 0, *p* <.001, *n* = 54). The median score for the upper, middle and lower image sections is 0.9, 0.94, and 0.75, respectively. The optimization thus substantially improves the results in all regions and all image depths.

### Brightness and quality degradation

**Fig 5:**
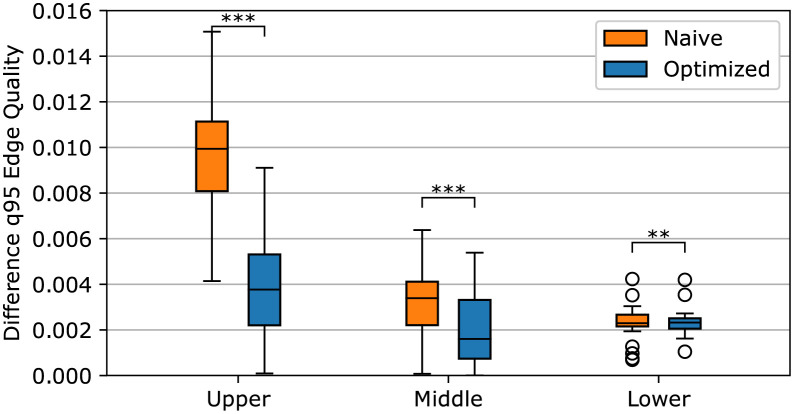
Difference in q95 edge quality between real and naive (orange bars, Naive) and real and optimized data (blue bars, Optimized). Four corresponding images are compared, and the results for the normalized z-indices are grouped into the upper, middle, and lower region.

For the comparison of brightness reduction and quality in occluded image regions between real, naive, and optimized images, the q95 measure is used. Fig 5 shows the results of the paired analysis between real and naive and real and optimized images. The results of four image pairs are grouped based on their normalized z-index. The groups are the upper, middle, and lower regions with the intervals of [0, 41), [41, 82), and [82, ∞) *px*, respectively.

The outcome shows that the difference between real and naive data gets smaller in deeper regions. While the upper region shows a median value of 0.01, the middle and lower regions have medians of 0.0033 and 0.0023, respectively. The interquartile range for the distance between real and naive data is also getting smaller for deeper image regions.

The analysis shows that the optimization improves the scores of the naive data. The optimized data reduces the difference to the real-world data by more than half compared to the naive data for the upper image region (median score 0.0037). The difference in the middle image region is still reduced by one-half compared to naive data but shows a greater overlap when comparing the interquartile distance. In the lower image region, the results of naive and optimized data are nearly identical. Based on the exact Wilcoxon test, the optimization significantly improves the results in all image regions (*w* = 13530, *p* <.001, *n* = 164∣*w* = 9123, *p* <.001, *n* = 149∣*w* = 417, *p* = .002, *n* = 32).

### Segmentation performance

**Fig 6:**
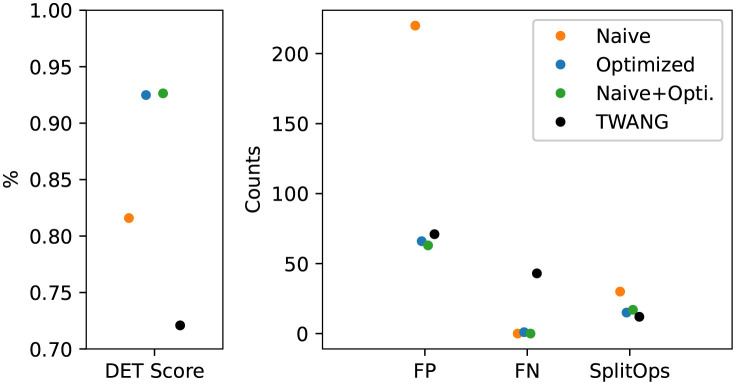
Performance of segmentation models on real data. The models are trained on naive and optimized image data. The left figure shows the detection accuracy (DET-Score), while the right figure shows the individual components of the metric: the number of false positive detections (FP), the number of false negative detections (FN), and the number of splitting operations (SplitOps).

Fig 6 shows the real data performance of segmentation models trained on the naive (*S*_naive_), optimized (*S*_opti_), and the combination of both data (*S*_naive+opti_). Additionally, the performance of the classical segmentation algorithm TWANG is given. The left graph shows the detection accuracy (DET), and the right graph shows the number of false positive and false negative detections and the number of required splitting operations.

The classical segmentation algorithm TWANG achieves a DET accuracy of 0.721 with 71 false positive, 43 false negative, and 12 missed splits. By using a deep learning segmentation model (see Methods/Segmentation) trained on only naive data (*S*_naive_), a DET accuracy of 0.815 can be achieved. The model *S*_naive_ makes 220 false positive, zero false negative detections, and 30 missed splits. Using optimized instead of naive data (*S*_opti_) increases the DET accuracy to a score of 0.925. The model *S*_naive_ gives 66 false positive, 1 false negative detections, and misses 15 splits. The combination of both naive and optimized data for training (*S*_naive+opti_) can only slightly increase the DET accuracy (0.926). The number of false positive and false negative detections is 63 and zero, respectively. The number of missed splits is 17.

## Discussion

The results of the Wasserstein metric show that for the region “background outside the spheroid”, the simulation is already able to produce data similar to the real images. This indicates that the simplified noise generation process is a good approximation to the real data, at least for this region. However, the analysis also shows that the simulation lacks realism within the cell culture. This matches the visual impression of the data shown in Fig 3, where naive data looks sharper and lacks additional noise present inside the cell culture. One explanation for this is the PSF used for the simulation. As the beads used to measure the PSF are only embedded in a homogeneous gel, the measurement process does not include the effect of abbreviations caused by the different components of a cell culture. Therefore, the measured PSF causes less spreading compared to the real PSF. Experiments were performed to embed fluorescent beads inside a cell culture, but due to high autofluorescence signals and clumping of beads, no valid measurement could be performed.

The Wasserstein metric shows that the optimization can improve the naive data in all image regions. As can also be seen in Fig 3, the greatest improvements are obtained in the regions within the cell culture. This indicates that the GAN can compensate the effects of the ideal PSF used in the simulation. Based on the image slices, the signal spreading introduced by the GAN seems not to be directly influenced by the brightness of the nuclei. The nuclei look equally sharp in the outer region and the darker inner region, which may be improved by using a space variable PSF during simulation. The variability could be coupled to the brightness reduction function and achieved by Gaussian filtering of the original PSF.

Importantly, the optimization seems to not change the shapes of the simulated nuclei. This is important, as otherwise the shapes of the labels would not match the image data. Especially Cycle-GAN-based synthesis methods, which directly transform ellipse-shaped label maps to image data, suffer from this problem. Additionally, based on visual comparison, no additional nuclei are placed or removed by the optimization. This indicates that the reduced domain gap by using simulated image data instead of label maps leads to better results.

The analysis of the edge degradation shows that the optimized images have a greater similarity to the real images than the naive images. This also corresponds to the visual impression (see Fig 3). Due to the low noise inside the spheroid and the reduced signal spreading, the edges in the naive image seem to be more pronounced compared to real and optimized images. However, by analyzing the raw values of the measure instead of their difference to the real data (see S1 Fig), it can be seen that the values of the naive data are consistently lower than those of the real data. This is in contrast to the aforementioned visual impression, as by the raw measure, the edges of the naive image are weaker. One explanation for this discrepancy is the generally lower brightness of the naive image compared to the real image. Interestingly, the optimization increases the intensity of the nuclei and thus also increases the edge strength. As the rate of reduction is nearly identical between naive and optimized data (see S1 Fig), it can be assumed that the optimization shows the same brightness reduction as the naive image but with a globally increased intensity. This means that no extra conditional channel featuring brightness information is required for the patch-based training and inference of the Cycle-GAN.

As depicted in Fig 5, the distance between the naive/optimized and real data decreases for deeper image regions due to the generally decreasing raw values of the measure. As the values get lower, the difference between them will also get smaller. This can be avoided by calculating the percentage deviation to the real data values.

**Fig 7:**
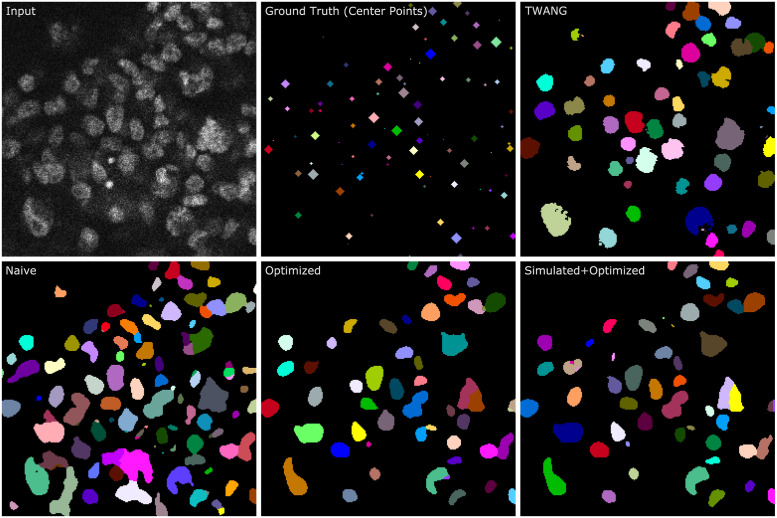
Zoomed 2D slices of a real image (upper left, Input), the corresponding ground truth (upper, middle, center point annotations) and segmentation results. As segmentation methods, the classical algorithm TWANG and deep learning algorithms trained with naive, optimized, and the combination are used as indicated. The center point annotations are enlarged by five repeated morphological dilation operations with a diamond shaped structuring element. The size of the center points therefore corresponds to their distance to the shown slice.

Based on the DET accuracy measure (Fig 7), the segmentation model trained only on naive data (*S*_naive_) can already outperform a classical segmentation algorithm on real image data. In contrast to the classical segmentation algorithm TWANG, the model *S*_naive_ delivers fewer false negative detections but considerably more false positive detections. As can be seen in Fig 7, *S*_naive_ often detects the background signal as foreground, which explains the high number of false positive detections. As the naive data does not feature realistic noise in background regions inside the spheroid, *S*_naive_ might not be able to distinguish high noise from the actual foreground signal. The seed detection threshold was reduced to reduce the high number of false positive detections of the TWANG algorithm, but this showed no improvement.

The performance of the deep learning segmentation model can be greatly increased by using optimized data instead of naive data (*S*_opti_), illustrating that the optimization is an important step in the pipeline. As can be seen in Fig 3, the optimized data shows an increased noise level inside the background region of the spheroid. In particular, this improves the number of false-positive detections since non-specific background signals are no longer recognized as foreground. (see Fig 7). Additionally, the use of optimized data reduces the number of merges, resulting in fewer required splitting operations.

The combination of naive data and optimized data for the training of the segmentation model (*S*_naive+opti_) only results in minimal improvements in the segmentation performance. The difference may be neglected as the performance is only measured on a single training run. The results of all segmentation models might be improved by hyperparameter optimization, as the parameters are set based on empirical values.

As shown, the method allows the generation of large 3D training datasets with minimal annotation effort, usable for the training of segmentation models. In this case, only ten nuclei were manually extracted from a high-resolution image. The otherwise required difficult, laborious, and very time-consuming annotation of 3D image data is exchanged with computation time and a one-time simulation parameter optimization.

With the augmentation and random placement of nuclei prototypes, a high variation in nuclei shape and positioning can be achieved. However, special variations, e.g., mitotic cells not being present in the nuclei database, will not be represented in the generated synthetic data. This can lead to segmentation errors if such objects are present in the images to be segmented. However, this also holds true for conventionally trained segmentation networks.

No special cell culture preparation is necessary to generate synthetic images with this method. While the proposed method is evaluated on images of spheroid cell culture models, other models, such as organoids or tissue samples, can also be synthesized. However, a limitation are distinct cell arrangements, e.g., present in the gut section, which cannot be replicated by the random nuclei positioning during the simulation. Furthermore, samples with only sliced nuclei, e.g., thin tissue sections, cannot be generated with this method. However, the annotation effort and complexity of such data is comparable to that of regular 2D images. Therefore, we think one is faster manually annotating this data than using the proposed synthesis method.

## Conclusion

In the past, conventional algorithms were first selected for segmenting new data, and then their parameters were optimized. With the increasing popularity of deep learning algorithms, the process changed and consisted of selecting a segmentation network and manually annotating a sufficient amount of training data. However, the possibility of creating realistic synthetic datasets again changes the process as the manual annotation can be skipped. Now a segmentation model is selected, and synthetic training data is generated.

In this work, we presented a new method for the synthesis of large 3D microscopic image data. We used a hybrid approach consisting of a simulation followed by a data-driven optimization. The simulation allows adjusting settings like cell density or the strength of the brightness decay. To feature a realistic brightness reduction in deeper image regions, we introduced a new reduction model which is based on the image content. With the introduced quality degradation measure, we could show that the new reduction model produces data with a similar decay compared to the real data.

Using the Cycle-GAN as a post-processing method, e.g., transforming simulated data to real data, we could show that no additional channel is required to introduce global properties like brightness distributions. The quantitative comparison of synthetic and real data showed that the deep-learning-based optimization overall improved the quality of the naive images, especially in the region inside the cell culture. Based on visual comparison, shape differences of nuclei between naive and optimized data are minimal, and neither objects are removed nor arbitrary ones inserted. We furthermore proved that the generated data can be used to train a deep learning segmentation model that can outperform a classical segmentation method.

In the future, we plan to use a physically motivated method for the placement of nucleus prototypes to achieve a more realistic orientation of nuclei and to directly obtain realistic cell cultures shapes. Furthermore, it is planned to investigate in the additional synthesis of nucleus prototypes to further increase the diversity.

## Supporting information

S1 Appendix Detailed pipeline description. (PDF)

S2 Appendix Image region definition. (PDF)

S1 Fig Raw values of the q95 edge degradation measure. (EPS)

S2 Fig Brightness reduction factor b calculated for different values of clearing parameter *p*. (EPS)
