# Supplementary material for: Synthesis of large scale 3D microscopic images of 3D cell cultures for training and benchmarking
Source: PLoS One. 2023 Mar 31;18(3):e0283828. doi: 10.1371/journal.pone.0283828 (PMC10065262; doi:10.1371/journal.pone.0283828)
Supplement: S1 Appendix — (PDF) [file pone.0283828.s001.pdf]

S1 Appendix

Synthesis of large scale 3D microscopic images of 3D cell cultures for training and benchmarking

Roman Bruch[1*], Florian Keller[2], Moritz Böhland[1], Mario Vitacolonna[2], Lukas Klinger[1], Rüdiger Rudolf[2], Markus Reischl[1]

1 Institute for Automation and Applied Informatics, Karlsruhe Institute of Technology, Eggenstein-Leopoldshafen, Germany
2 Institute of Molecular and Cell Biology, Mannheim University of Applied Sciences, Mannheim, Germany

Detailed Pipeline Description

Prototype generation

A cell culture sample is created during prototype generation by placing individual nucleus prototypes in an empty image. To achieve a realistic shape of the cell culture, a 3D shape mask, M_{shape}, can be used, thus limiting the possible placement positions. The mask can either be specified manually or by automatic segmentation of a real cell culture.

The nucleus prototype placement for the generation of the cell culture sample starts with the selection of a random nucleus prototype from the database. To increase the diversity, the selected prototype is augmented by mirroring and 90° rotations around the z-axis. Then, a random position is selected and checked for validity. If more than 50% of the nucleus volume is outside the shape mask, the placement process is restarted by selecting a new prototype. This is also the case if a nucleus overlaps a previously placed one by more than $t_{\text{overlap}}\%$ of its own volume. This threshold can be used to set the cell density of the generated sample. To also allow for lower cell densities, the nucleus to be placed can be enlarged for the duration of the overlap check. This is achieved by repeated binary dilation operations. The nucleus is then placed in its original size, resulting in a greater distance between the nuclei. The number of dilation operations is defined by the parameter $n_{dilation}$. If the validity checks are satisfied, the nucleus is placed at the selected position, and the placement process is repeated. If the validity checks have failed for i_{max} times in a row, the placement process is stopped, and the prototype generation is completed.

Imaging simulation

The imaging simulation involves simulating the effects caused by acquisition with a microscope. First, the brightness distribution within the sample is adjusted. Unlike other methods that use a function independent of the image content, we use a formula that depends on the shape of the generated cell culture.

For each voxel in the synthetic image, a brightness reduction factor $b(z, y, x)$ is calculated. This factor mainly depends on $i(z, y, x)$, the number of foreground voxel in z-direction above a position (z, y, x). The foreground regions are defined by the shape mask also used during the *prototype generation*. As different clearing methods can vary in effectiveness, the reduction can be tuned by the parameter p. In summary, the brightness reduction factor b is calculated as follows:

$$b(z, y, x) = max(min(\left(\frac{i(z, y, x)}{p} + 1\right)^{-6}, 1), 0). \quad (1)$$

The intensity of each voxel in the synthetic image is then multiplied by the brightness reduction factor b. S2 Fig shows the effect of different values of p on the reduction factor b.

After the brightness distribution is adjusted, the blur of the microscope optics is simulated. Ideally, the image formation process of the microscope is linear and shift-invariant and can thus be described entirely by the point spread function (PSF). The PSF represents the response of an imaging system to a point-shaped object, which is mathematically described by the convolution operation. In reality, mismatches of the refractive index inside a sample will result in a depth-dependent PSF. To model this effect, the simulation allows for the specification of multiple PSFs, which are then used to convolve overlapping depth regions of the image. To create smooth transitions between these regions, the values in the overlapping area are averaged with a distance-dependent influence.

The next part of the simulation focuses on the image sensor. The microscope provides a continuous signal, which is then discretized by the image sensor. The signal is already discrete in the simulation, but a similar effect can be achieved by downsampling the simulated image. The simulation can thus be run in a higher resolution which is then downsampled to the desired output resolution. The higher resolution also increases the quality of the convolution with the PSF, as the PSF is small in size.

After the downsampling, noise is added to the image. As the noise is complex and highly dependent on the detector and its measuring type, the introduction of noise into the simulated image is greatly simplified. Only a simple Poisson noise is calculated and applied to the simulated image, which relieves the user from the complex adjustment of various noise sources. The consecutive optimization step then performs the specific noise modeling corresponding to the real data.

Optimization

The goal of the optimization is to further reduce differences between real and simulated images. For this task, a Cycle-GAN is used as no paired training images are available.

The structure of the generator and discriminator networks are adopted from [1]. To process 3D image data, all 2D convolutions are replaced by 3D convolutions. The generator networks consist of one initial convolutional block, two convolutional blocks for downsampling, nine Resnet blocks [2], two transposed convolutional blocks for upsampling, and one final convolutional block. As discriminator network, a PatchGAN [3] is used. Due to the smaller patch size along the z-axis, convolutions are performed using a z-stride of one. The stride in the x- and y-axis are left unchanged.

3D GANs require large amounts of GPU memory, which makes a patch-based approach necessary to transform the large 3D images. A patch size of $32 \times 256 \times 256 px^3$ (z, y, x) is used for training and inference. As training data, four real and four naive images are used. In each epoch, four real and four naive training patches are generated by random cropping. Due to the 3D processing, a batch size of one is used. The discriminator networks are only trained every second epoch to stabilize the Cycle-GAN training. The Cycle-GAN is then trained for 7000 epochs on a *NVIDIA RTX A6000* graphics card with 48GB of memory. After 3500 epochs, the learning rate is reduced as proposed in [1]. The training took 145 h.

The simulated images with which the generator model was trained are also used for inference, as this can also be performed in a real use case. The simulated images were once again sliced into patches. For the inference, an overlap of 50% is used. This allows for better image reconstruction, as border regions of the patches can be removed. Each simulated patch was then transformed by the trained generator network. Afterwards, the patches are reassembled to form a complete image. As the patches were created with a 50% overlap, 25% is removed from each side of the patch before reconstruction.

References

1. Zhu JY, Park T, Isola P, Efros AA. Unpaired Image-to-Image Translation Using Cycle-Consistent Adversarial Networks. In: IEEE International Conference on Computer Vision (ICCV). IEEE; 2017.

2. He K, Zhang X, Ren S, Sun J. Deep residual learning for image recognition. In: Proceedings of the IEEE conference on computer vision and pattern recognition; 2016. p. 770–778.

3. Isola P, Zhu JY, Zhou T, Efros AA. Image-to-image translation with conditional adversarial networks. In: Proceedings of the IEEE conference on computer vision and pattern recognition; 2017. p. 1125–1134.
