# Supplementary material for: Synthesis of large scale 3D microscopic images of 3D cell cultures for training and benchmarking
Source: PLoS One. 2023 Mar 31;18(3):e0283828. doi: 10.1371/journal.pone.0283828 (PMC10065262; doi:10.1371/journal.pone.0283828)
Supplement: S2 Appendix — (PDF) [file pone.0283828.s002.pdf]

S2 Appendix

Synthesis of large scale 3D microscopic images of 3D cell cultures for training and benchmarking

Roman Bruch[1*], Florian Keller[2], Moritz Böhland[1], Mario Vitacolonna[2], Lukas Klinger[1], Rüdiger Rudolf[2], Markus Reischl[1]

1 Institute for Automation and Applied Informatics, Karlsruhe Institute of Technology, Eggenstein-Leopoldshafen, Germany
2 Institute of Molecular and Cell Biology, Mannheim University of Applied Sciences, Mannheim, Germany

Image region definition

For the calculation of the Wasserstein distance, the images are split into three regions: the background outside and within the cell culture, and the foreground. The following section describes the detailed procedure for the calculation of each region.

To calculate the background region inside the cell culture, 16 morphological dilation operations followed by 16 erosion operations are applied to the nuclei label mask. Afterwards, foreground regions of the initial label mask are defined as background. Two follow-up erosion operations ensure that foreground structures with fuzzy boundaries are entirely located outside the mask. The resulting mask is used as the background region inside the spheroid. For the calculation of the background region outside of the spheroid, a similar calculation is performed. After 16 dilation and 18 erosion operations, the remaining holes in the label mask are filled. Finally, the label mask is inverted to define the background as new foreground and vice versa. The resulting label mask defines the background region outside the spheroid. The foreground region inside the spheroid is defined by the nuclei label mask.
